# The Prevalence of Shoulder Pain and Awareness of Frozen Shoulder Among the General Population in Taif City, Saudi Arabia

**DOI:** 10.7759/cureus.58229

**Published:** 2024-04-14

**Authors:** Ahmed A Alghamdi, Mohammed H Alfaqih, Eyad H Alfaqih, Mohammed A Alamri, Layal H Alfaqih, Hussein H Mufti, Mohab S Almadani

**Affiliations:** 1 Orthopaedics, Alhada Hospital for Armed Forces, Taif, SAU; 2 General Practice, King Abdulaziz Specialist Hospital, Jeddah, SAU; 3 Orthopaedics, Alhada Armed Force Hospital, Taif, SAU; 4 Medicine and Surgery, Taif University, Taif, SAU; 5 Orthopaedics, King Abdulaziz Specialist Hospital, Jeddah, SAU

**Keywords:** taif, saudi arabia, awareness, prevalence, frozen shoulder, shoulder pain

## Abstract

Background

The global prevalence of shoulder pain varies widely across countries. Additionally, shoulder pain and frozen shoulder can significantly affect patients' quality of life due to high levels of pain and disability.

Objective

This study aimed to investigate the prevalence of shoulder pain and its risk factors. It also aims to assess the level of knowledge regarding frozen shoulders and its related factors in Taif City, Saudi Arabia.

Methods

A cross-sectional observational study was conducted in Taif City in December 2023 using a validated questionnaire comprised of socio-demographic characteristics, the prevalence of shoulder pain, and the awareness of frozen shoulders.

Results

A total of 378 participants enrolled in the study, with 54.8% being male and 62.7% being graduates and having jobs equally distributed among office (24.9%) and in the field (24.9%). Most participants were smokers (75.9%) and did not engage in body-building activities (79.6%). Around 26.5% of them had diabetes. The prevalence of shoulder pain was 32.8%. Aging from 35 to 44 years (p<0.001), having a higher salary from 6000 to 10000 SAR (p<0.001), retirement (p<0.001), engaging in body-building activities (p=0.035), having diabetes (p<0.001), and having other comorbidities (p<0.001) are significantly impacted having shoulder pain. Increased knowledge about the frozen shoulder is correlated with aging from 25-34 (p=0.026), smoking (p=0.002), engaging in bodybuilding (p<0.001), having diabetes (p=0.010), and having other medical conditions (p=0.010).

Conclusion

The study has shown that shoulder pain is prevalent among Taif City's population. Nevertheless, a low level of knowledge was observed. Therefore, enhancing the national educational programs is needed to increase public awareness of frozen shoulders.

## Introduction

Shoulder pain is a common problem worldwide that is prevalent among approximately 16% of the general population [1]. Frozen shoulder, also known as adhesive capsulitis, is a condition that causes pain and stiffness in the shoulder joint. It is characterized by severe pain and inability to move the shoulder. Frozen shoulders develop in three stages: freezing, frozen, and thawing. The condition usually improves within 12 to 18 months, but severe or persistent symptoms may require treatment [2].

The global prevalence and incidence of shoulder pain vary widely across countries. It is reported to be the third most common musculoskeletal symptom presenting for health care and makes up an estimated 4% of annual consultations by adults in UK primary care [3]. The prevalence of frozen shoulder is estimated to be between 2% and 5% of the general population globally [4]. Most patients are between 40 and 60 years old at diagnosis. However, some evidence suggests that frozen shoulders can occur later in life. Risk factors for developing frozen shoulders include diabetes, thyroid problems, hormone changes, shoulder injury, shoulder surgery, open heart surgery, and cervical disk disease of the neck [5].

In Saudi Arabia, the condition has been prominent in several areas. A study conducted in the Western region of Saudi Arabia discovered that the prevalence of frozen shoulders among diabetic patients was 31.6% [6]. Another retrospective study conducted in the Qassim region discovered that the prevalence rate of frozen shoulders was 13.2% [7].

Shoulder pain and frozen shoulders can have a significant impact on patients' quality of life as they may experience high rates of pain and disability compared to the general population [8]. Complications that may arise include residual shoulder pain and stiffness, humeral fracture, rupture of the biceps and subscapularis tendons, and labral tears [9]. The long-term effects of frozen shoulders vary depending on the individual and the severity of the condition, which may affect all facets of life, including work impact, sleep, personal hygiene, interpersonal relationships, and independence [10].

There is not enough recent or updated data about the condition's prevalence among the general population in Saudi Arabia and Taif City. Therefore, this study aimed to assess the prevalence of shoulder pain and its associated factors. Furthermore, it aims to discuss the knowledge level and its correlated factors with frozen shoulders awareness among the general population in Taif City, Saudi Arabia.

## Materials and methods

Study design and duration

A cross-sectional observational study was performed among the general population in Taif City, Saudi Arabia. The data was collected in December 2023. 

Study population

The study included a general population of 16 years or older living in Taif City who are willing to participate in the study.

Sampling technique 

A simple random sampling technique was used in this study to select the participants

Sample size calculation

The Raosoft sample size calculator (2004, Raosoft, Inc., Seattle) was used online to calculate the sample size. Based on a confidence level of 95%, a margin of error of 5%, and a maximum uncertainty of 50% for positive responses, a minimum of 377 participants should be included in this study.

Data collection method

Data was collected through a structured and self-administered electronic questionnaire distributed to adults in Taif City as a link to Google form using social media platforms (e.g., Twitter, Instagram, Linked-in, WhatsApp, etc.).

Data collection tool

The data collection tool was a structured questionnaire designed based on a previous study entitled "The Prevalence of Shoulder Pain and Awareness of Frozen Shoulder Among the General Population in Assir Region" [6]. The questionnaire was validated in the Assir Region in Saudi Arabia, and it included three sections: the first section comprised participants' characteristics, the second section focused on the habits and diseases of the participants, and the third section addressed knowledge about frozen shoulder. The demographics included age, gender, educational status, working style, and income. Habits and diseases included questions about smoking status, diabetes and comorbidities, and exercise activity. Knowledge about frozen shoulder included questions related to age groups, disease signs and symptoms, gender, risk factors, anxiety, and depression.

Pilot study

The questionnaire was administered to 10 respondents to ensure that it was easily understood and to estimate the time needed to fill it out. Data collected in the pilot study were not included in the statistical analysis. 

Data entry and analysis

The data was extracted and revised in an Excel sheet. Statistical analysis was conducted using the SPSS (IBM Corp. Released 2019. IBM SPSS Statistics for Windows, Version 26.0. Armonk, NY: IBM Corp). Categorical variables were expressed in numbers and percentages, while continuous variables were checked for normality. The Chi-square test was used to compare different variables with shoulder pain and awareness of frozen shoulders. The statistical significance was established by considering p-values below 0.05.

Ethical considerations

Approval was given by the Research and Ethics Committee of Armed Forces Hospitals, with the application number 2023-822. 

## Results

The study included 378 participants, with about half (185, 48.9%) aged 45 or older (207, 54.8%) male. The majority were graduates (237, 62.7%). Approximately half of them had an income of more than 10,000 Saudi Riyal (SAR) per month(186, 49.2%). About a quarter of the participants were unemployed (94, 24.9%), worked in office-based jobs (94, 24.9%), and worked in field-based jobs (94, 24.9%). Full details are shown in Table 1.

**Table 1:** Demographic characteristics of the participants (N=378) SAR: Saudi Riyal.

Parameters	Category	Number (N)	Percentage (%)
Age (Years)	16-24	54	14.3
	25-34	56	14.8
	35-44	83	22.0
	More than or equal to 45	185	48.9
Gender	Male	207	54.8
	Female	171	45.2
Income (SAR)	Less than 6000	94	24.9
	From 6000 to 10000	98	25.9
	More than 10000	186	49.2
Educational level	Uneducated	9	2.4
	High school student	77	20.4
	College student	55	14.6
	Graduated	237	62.7
Working style (N=377)	Unemployed	94	24.9
	Lawyer	1	0.3
	Teacher	6	1.6
	Field-based	94	24.9
	Office-based	94	24.9
	Pharmacist	1	0.3
	Military	2	0.5
	Private sector	1	0.3
	School agent	2	0.5
	Retired	82	21.7

Regarding respondents' habits and disease, most participants were smokers (287, 75.9%) and did not practice bodybuilding (301, 79.6%). About a quarter of them had diabetes (100, 26.5%), with 80 patients (80%) having type II diabetes, and 33 cases (33%) had diabetes for more than ten years. More details are presented in Table 2.

**Table 2:** Habits and diseases of the participants (N=378)

Parameters	Category	Number (N)	Percentage (%)
Smoking	Yes	287	75.9
	No	91	24.1
Practicing bodybuilding	Yes	77	20.4
	No	301	79.6
Having diabetes	Yes	100	26.5
	No	278	73.5
Type of diabetes (N=100)	Type I	20	20
	Type II	80	80
Duration of having diabetes (N=100)	Less than one year	6	6
	From 1 to 5 years	30	30
	From 5 to 10 years	31	31
	More than 10 years	33	33
Having other comorbidities	Yes	128	34
	No	250	66
If yes, please specify (N=128)	Hypertension	41	32.0
	Joint diseases	35	27.3
	Glands disorder	25	19.5
	Kidney diseases	13	10.2
	Heart diseases	13	10.2
	Psychiatric illness	8	6.3
	Asthma	3	2.3
	Anemia	3	2.3
	Problems in vertebras	2	1.6
	Others	6	4.7

**Figure 1:**
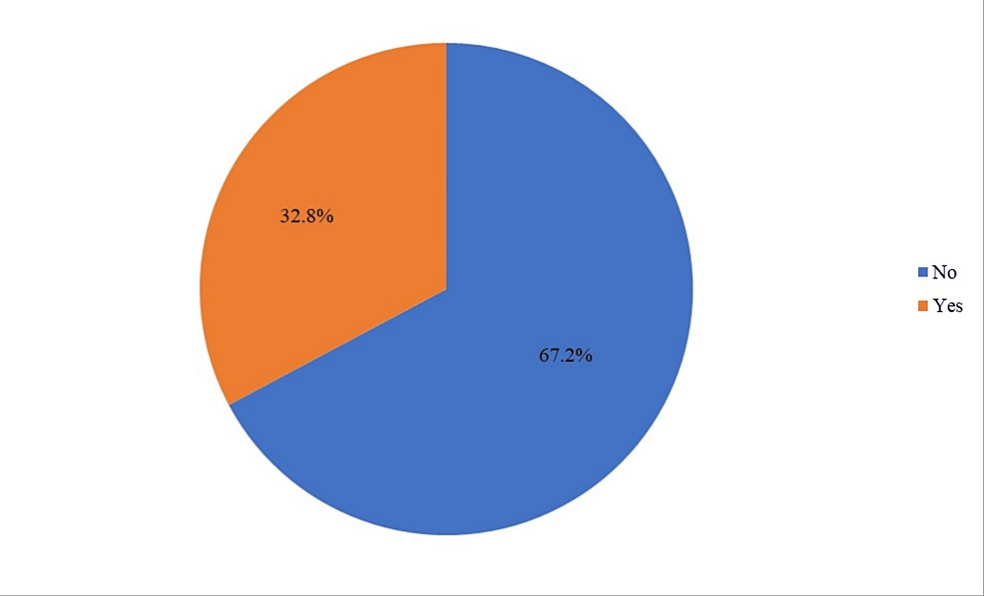
Prevalence of shoulder pain in Taif City

As illustrated in Figure 1, the prevalence of shoulder pain in Taif City was (124, 32.8%).

Table 3 illustrates the participants' knowledge about frozen shoulder. Most of them did not know frozen shoulders (321, 84.9%). More than half of the participants reported that individuals aged between 40 and 60 are the most affected by the frozen shoulder (208, 55%); also, (217, 57.4%) reported all the signs and symptoms of a frozen shoulder. Also, the most reported risk factors for a frozen shoulder were immobilization for the long term (182, 48.1%), followed by diabetes mellitus and shoulder trauma (155, 41%). Finally, anxiety (167, 44.2%) and depression (134, 35.4%) were reported to increase the risk of a frozen shoulder.

**Table 3:** Respondents' knowledge about frozen shoulder

Factors	Categories	Number (N)	Percentage (%)
knowledge about frozen shoulder	Yes	57	15.1
	No	321	84.9
Age groups that are most commonly affected by frozen shoulder	Less than 20 years	15	4
	20 – 40 years	72	19
	40 – 60 years	208	55
	More than 60 years	83	22
Signs and symptoms of a frozen shoulder	Shoulder pain	111	29.4
	The shoulder's range of motion starts decreasing	67	17.7
	Stiffness	35	9.3
	Shoulder swelling	20	5.3
	All	217	57.4
Gender is more likely to be affected by a frozen shoulder	Male	188	49.7
	Female	190	50.3
Risk factors for a frozen shoulder	Immobilization for long-term	182	48.1
	Diabetes mellitus	155	41
	Shoulder trauma	155	41
	Thyroid disease	65	17.2
	Hyperlipidemia	44	11.6
	Autoimmune diseases	38	10.1
Depression increases the risk of getting a frozen shoulder	Yes	134	35.4
	No	244	64.6
Anxiety increases the risk of getting a frozen shoulder	Yes	167	44.2
	No	211	55.8

Factors correlated with shoulder pain were assessed in Table 4. Shoulder pain has a significant correlation with age (p<0.001), income (p<0.001), and working style (p<0.001) of the participants. A higher prevalence of shoulder pain was observed in participants aged 35 to 44 years, with income from 6000 to 10000 SAR, and retired participants. However, the gender and education level of the participants showed no significant correlation with shoulder pain. 

**Table 4:** Correlation between the shoulder pain and respondents' characteristics: N: number, SAR: Saudi Riyal. p-value <0.05 is considered statistically significant.

Factors		Shoulder pain N (%)		p-value
		Yes	No	
Age (Years)	16-24	4 (7.4)	50 (92.6)	<0.001
	25-34	18 (32.1)	38 (67.9)	
	35-44	35 (42.2)	48 (57.8)	
	More than or equal to 45	67 (36.2)	118 (63.8)	
Gender	Male	63 (30.4)	144 (69.6)	0.280
	Female	61 (35.7)	110 (64.3)	
Income (SAR)	Less than 6000	17 (18.1)	77 (81.9)	<0.001
	From 6000 to 10000	45 (45.9)	53 (54.1)	
	More than 10000	62 (33.3)	124 (66.7)	
Educational level	Uneducated	5 (55.6)	4 (44.4)	0.469
	High school student	23 (29.9)	54 (70.1)	
	College student	17 (30.9)	38 (69.1)	
	Graduated	79 (33.3)	158 (66.7)	
Working style	Unemployed	19 (20.2)	75 (79.8)	<0.001
	Office-based	36 (34.3)	69 (65.7)	
	Field-based	27 (28.1)	69 (71.9)	
	Retired	42 (51.2)	40 (48.8)	

Additionally, regarding individuals' habits and comorbidities (Table 5), practicing bodybuilding [odds ratio(OR)=1.42, p=0.035], having diabetes (OR=1.88, p<0.001), and having comorbidities (OR=2.27, p<0.001) showed a significant correlation with shoulder pain. However, smoking status showed no significant correlation with shoulder pain.

**Table 5:** Association between shoulder pain and the habits and diseases of the participants N: number, p-value <0.05 is considered statistically significant.

Factors		Shoulder pain N (%)		p-value
		Yes	No	
Smoking	Yes	33 (36.3)	58 (63.7)	0.420
	No	196 (68.3)	91 (31.7)	
Doing sports such as bodybuilding	Yes	33 (42.9)	44 (57.1)	0.035
	No	91 (30.2)	210 (69.8)	
Having diabetes	Yes	50 (50)	50 (50)	<0.001
	No	74 (26.6)	204 (73.4)	
Having other comorbidities	Yes	67 (51.9)	62 (48.1)	<0.001
	No	57 (22.9)	192 (77.1)	

Concerning associated factors with increased awareness of frozen shoulder (Table 6), no significant correlation was seen between the individuals' characteristics and shoulder pain except for age (P=0.026). The participants aged 25 to 34 years (13, 23.2%) had the highest knowledge about frozen shoulders.

**Table 6:** Correlated factors associated with knowledge about frozen shoulder p-value <0.05 is considered statistically significant.

Factors		Knowledge about frozen shoulder N (%)		p-value
		Yes	No	
Age (Years)	16-24	7 (13)	47 (87)	0.026
	25-34	13 (23.2)	43 (76.8)	
	35-44	18 (21.7)	65 (78.3)	
	More than or equal to 45	19 (10.3)	166 (89.7)	
Gender	Male	34 (16.4)	173 (83.6)	0.421
	Female	23 (13.5)	148 (86.5)	
Income (SAR)	Less than 5000	12 (12.8)	82 (87.2)	0.228
	From 6000 to 10000	20 (20.4)	78 (79.6)	
	More than 10000	25 (13.4)	161 (86.6)	
Educational level	Uneducated	3 (33.3)	6 (66.7)	0.492
	High school student	11 (14.3)	66 (85.7)	
	College student	8 (14.5)	47 (85.5)	
	Graduated	35 (14.8)	202 (85.2)	
Working style	Unemployed	17 (18.1)	77 (81.9)	0.815
	Office-based	14 (13.3)	91 (86.7)	
	Field-based	14 (14.6)	82 (85.4)	
	Retired	12 (14.6)	70 (85.4)	

Regarding the impact of habits and having comorbidities on frozen shoulder knowledge (Table 7), a significant correlation between being a smoker (OR=2.14, p=0.002), practicing bodybuilding (OR=2.47, p<0.001), having diabetes (1.89, p=0.010), and having comorbidities (OR=1.87, p=0.010) with higher knowledge about frozen shoulder.

**Table 7:** Association between the habits and diseases of the participants and knowledge about frozen shoulder p-value <0.05 is considered statistically significant.

Factors		Knowledge about frozen shoulder N (%)		p-value
		Yes	No	
Smoking	Yes	23 (25.3)	68 (74.7)	0.002
	No	34 (11.8)	253 (88.2)	
Practicing bodybuilding	Yes	22 (28.6)	55 (71.4)	<0.001
	No	35 (11.6)	266 (88.4)	
Having diabetes	Yes	23 (23)	77 (77)	0.010
	No	34 (12.2)	244 (87.8)	
Having other comorbidities	Yes	28 (21.7)	101 (78.3)	0.010
	No	29 (11.6)	220 (88.4)	

## Discussion

This study aimed to assess the prevalence of shoulder pain and awareness of frozen shoulders among the general population in Taif City, Saudi Arabia. The prevalence of shoulder pain in Taif City was (124, 32.8%). Shoulder pain significantly correlated with age, income, working style of the participants, practicing bodybuilding, diabetes, and comorbidities. Additionally, age, smoking status, bodybuilding, diabetes, and comorbidities showed a significant correlation with knowledge about frozen shoulders.

According to our study, shoulder pain in Taif City, Saudi Arabia, was (124, 32.8%), similar to Japan's (30%) [11]. However, the prevalence in Taif City was higher than in the urban areas of Bauru [12](24%) and the urban regions of India (2%), as well as in rural areas of India (7.4%) [13]. Conversely, the prevalence in Taif City was lower than in the Netherlands [14](48%) and China [15] (48.7%). These variations in prevalence rates may be because there is no uniform definition for the clinical condition and anatomical area of the cervical and shoulder regions across different studies [12].

Our research indicated that shoulder pain is commonly experienced by individuals aged 35-44 years, 45 years or older, and those who retire (above 60 years of age). These findings are similar to studies conducted in Bauru [13] and the Netherlands [16], which also reported that shoulder pain was linked with people aged 60 years or older. However, shoulder pain was observed more frequently in Japan in young adults [16]. The increased prevalence of shoulder pain in older adults can be attributed to degenerative changes in muscles, tendons, ligaments, and joints that naturally occur with aging, chronic overload for the elderly worker, and prolonged exposure to occupational risk factors [16].

Unfortunately, (321, 84.9%) of individuals in Taif did not know frozen shoulders. However, in a previous study in Aseer in Saudi Arabia, 69.31% had sufficient knowledge about frozen shoulders [6]. Regarding the symptoms of frozen shoulders, the Aseer region study [6], reported that about half believed that multiple symptoms aligned with our study, where most participants reported all symptoms (shoulder pain, start decreasing shoulder range of motion (ROM), stiffness, and swelling). Additionally, the most reported risk factors in our study for a frozen shoulder were immobilization for the long term, diabetes mellitus, thyroid diseases, and shoulder trauma, which is per the published literature [17,18]. In our study, a low percentage agreed that anxiety and depression increase the risk of a frozen shoulder, which was in contrast to Aseer's study, which demonstrated that a much higher percentage of individuals thought that depression and anxiety could increase the chance of shoulder diseases [6].

 In our study, age was a significant factor impacting the awareness about frozen shoulders in Taif City, where the participants aged from 25 to 34 years had higher knowledge. However, the Aseer region study [6]. reported that gender was a significant factor in awareness about frozen shoulders. It could return to the culture of regions in Saudi. Additionally, smoking, practicing bodybuilding, having diabetes, and other comorbidities had a higher knowledge about frozen shoulders than others. It could return to the possibility of having frozen shoulders with such factors.

Limitations

These results were obtained only from Taif City and cannot be generalized to the entire Saudi population. More extensive studies may help better understand the factors influencing the prevalence of shoulder pain and awareness about frozen shoulders in Saudi Arabia.

## Conclusions

Our findings have shown widespread shoulder pain among the general population in Taif City. Certain habits and comorbidities had an impact on the prevalence of shoulder pain. Additionally, most of Taif's population had a low awareness of frozen shoulders. Therefore, it is essential to provide campaigns to increase awareness about frozen shoulders, their symptoms, habits, and diseases affecting shoulder pain.
